# Volunteerism as a multidimensional form of human prosocial engagement

**DOI:** 10.3389/fpubh.2026.1908108

**Published:** 2026-08-18

**Authors:** Orly Sarid

**Affiliations:** 1 The Spitzer Department of Social Work, Ben-Gurion University of the Negev, Beer-Sheva, Israel; 2 Goldman Sonnenfeldt School of Sustainability and Climate Change, Ben-Gurion University of the Negev, Beer-Sheva, Israel

**Keywords:** burnout, high-cost helping, moral identity, multidimensional framework, prosocial behavior, volunteer wellbeing, volunteerism

## Abstract

Volunteerism is widely recognized as an important contributor to public health, community resilience, and social cohesion, yet its conceptualization remains fragmented across disciplines. Existing research has advanced understanding of volunteer motivations, identity processes, wellbeing outcomes, and organizational influences, but these perspectives are typically examined separately rather than integrated within a common framework. This paper proposes a multidimensional framework that conceptualizes volunteerism as a form of prosocial behaviors defined by seven dimensions: motivational basis (D1), personal cost (D2), social embeddedness (D3), identity integration (D4), moral commitment (D5), meaning (D6), and psychological consequence (D7). The dimensions are conceptualized as distinct but correlated, with forms of helping characterized by their profiles across all seven. The framework distinguishes volunteerism from altruism by treating volunteerism as a behavioral form of helping that may arise from diverse motivations, whereas altruism is conceptualized as one possible motivational orientation. Drawing on research from public health, psychology, sociology, and moral cognition, the paper situates informal helping, structured volunteering, and high-cost helping within a single multidimensional space. Attention is given to the conditions under which volunteering is associated with wellbeing, resilience, social connectedness, and purpose, as well as to circumstances in which emotionally demanding or poorly supported roles may contribute to compassion fatigue, emotional exhaustion, and burnout. The framework advances a prospectively testable, configural account of high-cost helping, proposing that such acts are characterized by the joint elevation of personal cost, moral commitment, identity integration, and meaning, together with low anticipated reward. Five propositions and a corresponding measurement program are specified to guide empirical evaluation. By integrating motivations, costs, meanings, contexts, and consequences within a single model, the framework provides a foundation for future research and informs public-health and organizational strategies aimed at supporting both community benefit and volunteer wellbeing.

## 1 Introduction

This paper develops a multidimensional framework of volunteerism that conceptualizes volunteerism as a seven-dimensional behavioral construct. Volunteerism occupies an increasingly important place in contemporary societies, contributing to health promotion, social welfare, education, environmental action, humanitarian assistance, community development, civic participation, and disaster response. Yet volunteer activities vary substantially in motivation, intensity, duration, organizational context, personal cost, and psychological consequences. Existing theories have generated important insights into why people volunteer, how volunteer identities develop, and what sustains participation over time. However, they offer limited means of describing volunteerism itself as a multidimensional behavioral phenomenon or systematically comparing different forms of volunteer engagement within a common conceptual structure.

The need for such a framework is reinforced by several practical and scientific considerations. Volunteers constitute a substantial social resource whose contributions increasingly support institutions, communities, and public services across multiple sectors. Volunteering may generate important psychological, social, and health benefits, including enhanced wellbeing, meaning, social connectedness, and civic engagement. At the same time, volunteer activities may involve considerable personal costs, including emotional burden, stress, role strain, and burnout, particularly in demanding settings such as caregiving, disaster response, humanitarian assistance, and crisis intervention. Growing reliance on volunteer labor has also raised questions about the boundary between complementary civic action and the substitution of unpaid labor for formal services. Understanding these diverse forms and consequences of volunteer engagement requires a framework capable of integrating both beneficial and burdensome aspects of participation.

Volunteerism is generally defined as the voluntary contribution of time, effort, skills, knowledge, or resources for the benefit of individuals, communities, or causes without direct material compensation (1–4). Although volunteer activity may occur through formal organizations or independent initiatives, it is typically understood as a socially meaningful and relatively structured form of prosocial behavior directed beyond immediate personal or familial interests.

Several influential frameworks have advanced understanding of volunteer behavior. These include the functional approach to volunteer motivation (5, 6), Self-Determination Theory (7), role identity theory (8), volunteer engagement frameworks (9), and the volunteer process model of Omoto and Snyder (10). At a broader level, Penner et al. (11) conceptualize prosocial behavior across multiple levels of analysis, while Pfattheicher et al. (12) distinguish pro-sociality and altruism according to intention, consequences, and social evaluation. These approaches have substantially advanced understanding of volunteer behavior and, in several cases, provide explicitly integrative accounts.

Recent studies by Aslan and Tuncay strengthen this point by showing that volunteer engagement is shaped simultaneously by motives, barriers, perceived effects, social perceptions, organizational conditions, and broader societal context, including in child-welfare volunteering and active-volunteer samples (13–15). In the present framework, these findings support a multidimensional approach rather than adding a separate dimension.

The contribution of the present framework is not to replace these approaches but to address a different conceptual problem. Existing theories primarily explain why people volunteer, how participation is sustained, or how volunteer identities develop. The framework proposed here instead conceptualizes volunteerism itself as a multidimensional behavioral construct. Specifically, it distinguishes volunteer behavior from volunteer motivation, incorporates personal cost and psychological consequences as intrinsic dimensions of volunteer engagement rather than merely downstream outcomes, and provides an empirically testable structure for comparing diverse forms of volunteer activity, including high-cost helping. Section 2 develops this multidimensional construct in detail.

## 2 A multidimensional framework of prosocial engagement

This section defines the framework as a multidimensional construct. Prosocial engagement is conceptualized as a position within a seven-dimensional space. Each dimension is operationally defined, distinguished from closely related concepts, and later linked to candidate indicators in Section 7. Together, the seven dimensions constitute the full framework. Any shorter list used elsewhere in the paper is intended only as shorthand, not as an alternative dimensional structure. The seven dimensions were developed through conceptual synthesis, rather than as the product of an exhaustive systematic review. The synthesis draws on recurrent explanatory domains in the volunteerism and prosocial-behavior literatures: motives and functional accounts of volunteering (D1), cost and demand models of helping (D2), organizational mediation and role context (D3), role and social identity (D4), moral identity and values (D5), existential and meaning-making accounts (D6), and the wellbeing and burnout literature on the consequences of helping (D7). The framework therefore does not claim that these are the only possible attributes of volunteer activity. Rather, it identifies a minimal integrative set of dimensions needed to connect bodies of literature that are often examined separately.

Alternative candidate dimensions were treated as either antecedents, moderators, indicators, or subcomponents rather than independent dimensions. For example, culture, age, gender, socioeconomic resources, and prior experience are expected to shape where a person is located in the space; role duration, intensity, and exposure level are indicators of D2 and D3; organizational support is treated mainly as a moderator of the D2–D7 relationship; and recipient type or cause area is a contextual specification rather than a separate axis. This derivation is now made explicit so that the framework is not read as an arbitrary list.

### 2.1 The seven dimensions

**D1. Motivational basis:** The constellation of motives that initiates and sustains prosocial engagement, ranging from other-oriented (altruistic) concerns to social, identity-related, instrumental, and self-development motives. *Discriminant note:* D1 concerns why individuals are moved to help, whereas D5, Moral Commitment, concerns whether helping is experienced as a moral obligation. Thus, a person may be highly motivated to help without experiencing a strong sense of duty or may feel morally obligated to help regardless of the specific motives involved. Within D1, anticipated reward is treated as a prospective expectancy rather than as an outcome. It refers to the degree to which the actor expects personal benefit before or during action, such as social approval, gratitude, recognition, career benefit, status, emotional satisfaction, or reciprocal support. This makes it distinct from D7, which concerns the actual psychological consequence after engagement, and from D3, which concerns the social or organizational structure in which the act occurs. Empirically, D1 can therefore be modeled as a multi-facet domain including other-oriented concern, social-recognition motives, instrumental or self-development motives, and expected affective reward. The claim that high-cost helping involves low anticipated reward refers specifically to low expected personal benefit, not to the absence of meaning, moral obligation, or social embeddedness.

**D2. Personal cost:** The magnitude of resources expended, and risks incurred in the course of prosocial engagement, including investments of time, effort, money, emotional labor, and exposure to physical danger. *Discriminant note:* D2 concerns what is given up, expended, or borne in helping. It is conceptually distinct from D7 (Psychological Consequence), which concerns the psychological impact of bearing those costs. Comparable levels of personal cost may be associated with either growth and fulfillment or stress and depletion, depending on the availability of personal, social, and organizational resources.

**D3. Social embeddedness:** The degree to which the prosocial act is organized within, and mediated by, social structures, roles, and relationships, ranging from spontaneous individual helping to formally coordinated organizational service. *Discriminant note:* D3 concerns the structural context in which the act is carried out. It is distinguished from D4, Identity Integration, which concerns the act's place within the self. The two dimensions are conceptually distinct: highly embedded organizational roles may remain peripheral to identity, whereas deeply identity-central helping may occur outside formal roles or organizations. D3 is not intended to collapse volunteering into all prosocial behavior. Rather, it specifies the degree of organizational and relational mediation through which a helping act is carried out. Informal helping occupies the low-D3 boundary region of the space, where the act is typically carried out outside formal roles, organizations, or coordinated civic initiatives. Formal volunteering occupies the high-D3 region because it is connected to a role, organization, collective project, or coordinated civic initiative. Thus, the framework can compare informal helping and formal volunteering within a shared conceptual space without treating them as identical.

**D4. Identity integration:** The extent to which helping is central to the individual's self-concept and to membership in a valued group. *Discriminant note*: D4 concerns the centrality of helping to the self. It is distinguished from D6, Meaning, in that an activity may be identity-central yet experienced as routine, or personally meaningful yet not self-defining. D4 is also developmental rather than static. Volunteer identities may emerge through repeated participation, role learning, organizational socialization, and transitions across stages of involvement. The volunteer stages and transitions model, for example, describes movement from nominee and newcomer positions through emotional involvement and established volunteering to retirement from the role (16). Similarly, biographical and volunteer-process approaches treat volunteering as embedded in the life course, in role continuity, and, in some cases, in a sustained calling, rather than merely as a momentary self-description (10, 17). In the present framework, these volunteer-career processes are located primarily in D4, while their organizational conditions are captured by D3 and their motivational changes by D1. Identity theory further clarifies D4 by distinguishing the salience of a volunteer role within a person's role hierarchy from the broader moral or existential meaning of the activity (18). In this sense, D4 can be measured as role identity in ordinary volunteering and as stronger social or fused identity in high-cost helping.

**D5. Moral commitment:** The strength of internalized moral obligation and values experienced as binding on action, largely independent of anticipated reward. *Discriminant note:* D5 concerns obligation and values; it is distinguished from D1 (motivational basis) in that obligation is a felt imperative rather than a motive among others, and from D4 in that one may feel a moral duty to help strangers without that duty being identity-defining.

**D6. Meaning:** The degree to which the act is experienced as significant, purposeful, and coherent within the individual's wider understanding of a worthwhile life. *Discriminant note:* D6 concerns experienced significance and purpose [the will to meaning in the sense of Frankl (19), and the meaning-making of Park (20)]; it is distinguished from D4 (identity) in that meaning is about purpose and coherence rather than self-definition, and from D5 in that meaning need not be felt as obligation.

**D7. Psychological consequence:** This dimension refers to the effects that prosocial engagement has on the individual's psychological wellbeing. These effects may be positive, negative, or mixed. At one end, volunteering or helping others may contribute to personal growth, a stronger sense of connectedness, and a clearer sense of purpose. At the other end, the same kind of engagement may lead to stress, compassion fatigue, emotional exhaustion, or burnout. D7 is the only dimension in the framework that focuses on outcomes. It should therefore be distinguished from D2, Personal Cost. Personal cost refers to the demands or sacrifices associated with the act itself, such as time, effort, risk, or emotional burden. Psychological consequence refers to how the individual is affected by these demands over time. Two individuals may experience similar personal costs, yet the psychological consequences may differ greatly depending on the support they receive, the meaning they attach to their engagement, and the personal or social resources available to them.

Including D7 in the framework does not mean that psychological consequence is part of the minimal definition of volunteering. In other words, an act does not have to produce a particular psychological outcome in order to count as volunteering. Rather, D7 is included because the framework is not limited to identifying whether an act is volunteering. It also aims to describe and compare volunteer engagement as an experienced, relational, and socially supported process. In longitudinal research, D7 should be treated as an outcome that develops over time. It can be modeled as the result of the preceding configuration of D1–D6. At the same time, it remains one of the seven dimensions needed to provide a full analytic profile of volunteerism.

### 2.2 How the dimensions combine

**Figure 1:**
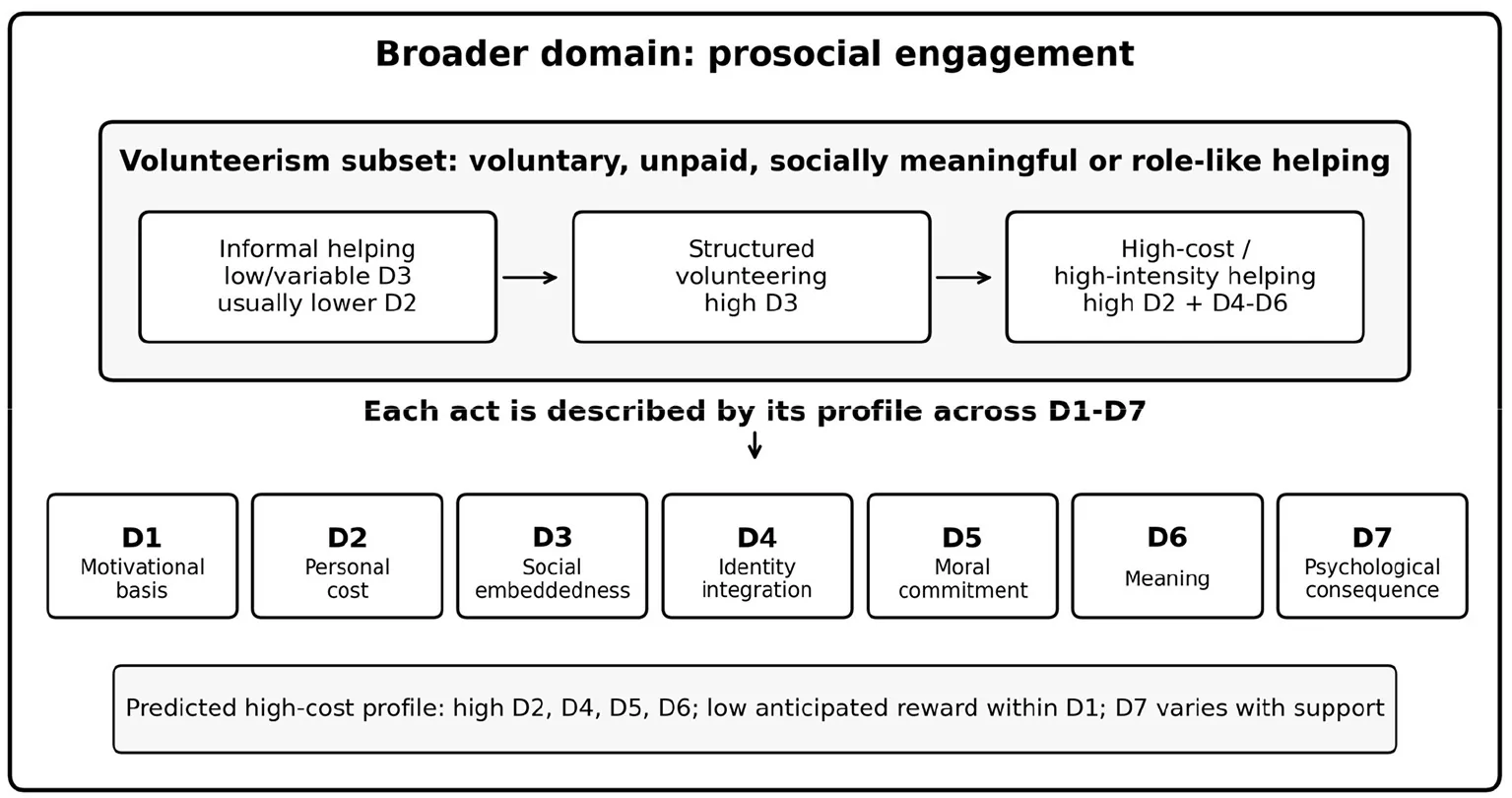
Schematic representation of the seven-dimensional framework. Prosocial engagement is the broader domain; volunteerism is located within it as voluntary, unpaid, socially meaningful or role-like helping. Informal helping, structured volunteering, and high-cost helping are shown as regions rather than fixed categories. Each act is described by its profile across D1-D7; high-cost helping is predicted by high D2, D4, D5, and D6 together with low anticipated reward within D1, while D7 varies with support.

An act is defined by its profile, the joint pattern of values across D1 to D7, not by a single summary score and not by any one dimension. Two acts with identical total intensity may occupy very different regions of space, and it is the configuration, not the sum, that carries the framework's explanatory claims. This configural commitment is what makes the account of high cost helping in Section 4 falsifiable: a specific profile is predicted there, shown in the high-cost column of Table 1, and a different observed profile would disconfirm it. Figure 1 depicts this space schematically.

**Table 1:** Dimensional profiles of ideal-typical forms of prosocial engagement.

Dimension	Informal helping	Structured volunteering	High-cost/high-intensity helping
D1 Motivational basis	Mixed; often situational, low anticipated reward salience	Mixed; social, identity, and instrumental motives common	Other-oriented; anticipated reward low
D2 Personal cost	Limited or situational	Moderate and sustained	Substantial; physical and emotional, may include risk to life
D3 Social embeddedness	Variable; often spontaneous	High; organizationally mediated	Variable; may be organized or spontaneous
D4 Identity integration	Variable	High	Very high
D5 Moral commitment	Low to moderate	Moderate	Very high; experienced as binding
D6 Meaning	Variable	High	Very high
D7 Psychological consequence	Usually positive; low burden	Positive if supported; burnout risk if unsupported	Polarized; growth or moral injury depending on support

Rows are the seven canonical dimensions (Section 2); Entries give the predicted level of each dimension.

The seven dimensions are intended to be conceptually distinct but empirically correlated, and the set is proposed as a working basis for measurement rather than as an exhaustive partition of all prosocial variation; it is, in that sense, a defined but revisable set rather than a closed taxonomy. The revisability and the seven-factor commitment of Proposition 1 (Section 7) are reconcilable rather than at odds: the set may be revised across studies as evidence accumulates, but for any given test Proposition 1 commits to exactly seven factors as the *a priori* model, and it is precisely the disconfirmation of that seven-factor model (for example a merged identity-meaning factor) that would trigger revision of the set. Section 7 specifies how the distinctness claim can be confirmed or disconfirmed.

### 2.3 What the framework adds over existing models

Table 2 contrasts the framework with the models the paper builds on. The functional approach (5) specifies why people volunteer (a theory of D1) but does not represent cost, consequence, or the configuration of high-cost acts. Self-Determination Theory (7) explains how autonomous motivation is sustained through autonomy, competence, and relatedness, which map onto aspects of D1, D3, and D6, but is silent on personal cost and on the moral-obligation pole of D5. Role identity theory (8) addresses D4 for ordinary role-based volunteering but not the social-identity, self-group mechanisms that the self-sacrifice literature requires. Penner's interactionist model (3) and the multilevel perspective of Penner et al. (11) integrate dispositional and organizational influences across levels, but they treat outcomes as downstream rather than as a constitutive dimension and do not generate a configural prediction for extreme helping. Pfattheicher et al. (12) supply the outcome/motive/norm taxonomy that this paper uses as an analytic instrument, but theirs is a typology of definitions rather than a measurement space. The framework adds a constitutive cost-and-consequence axis and a configural, falsifiable account of high-cost helping that the cited models, each strong on a subset of the dimensions, do not jointly provide. This is consistent with the D2 vs. D7 discriminant note. Cost concerns what is endured, whereas consequences concern how bearing that cost turns out. Consequently, two episodes may involve identical costs but very different psychological outcomes, demonstrating that D2 and D7 capture distinct dimensions of engagement. Table 1 shows the dimensional profiles that carry this claim. In response to the volunteer-career literature, the framework treats identity integration as a process that unfolds over time. Volunteer-process and stages-and-transitions models explain recruitment, socialization, maintenance, and exit; the present framework adds a way to locate these processes within a broader profile that includes cost, moral commitment, meaning, social embeddedness, and psychological consequence. This distinction clarifies that D4 includes both role identity at a given point in time and the longer developmental trajectory through which volunteering may become a life pathway.

**Table 2:** Contribution of the framework relative to cited models.

Model	Primarily captures	What the framework adds
Functional approach (5)	Why people volunteer (motives; D1)	Cost (D2), consequence (D7), and a configural high-cost account
Self-Determination Theory (7)	How autonomous motivation is sustained (aspects of D1, D3, D6)	Personal cost and the moral-obligation pole of D5; configural prediction
Role identity theory (8)	Role-based identity in ordinary volunteering (D4)	Social-identity and fusion mechanisms for self-sacrifice; full dimension set
Penner interactionist/multilevel (3, 11)	Dispositional and organizational influences across levels	Outcome as a constitutive dimension (D7); falsifiable configural profile
Prosociality typology (12)	Outcome/motive/norm definitions of altruism	A measurement space and configural account that operationalizes the typology
Volunteer process/stages and transitions models (10, 16)	How volunteering unfolds across recruitment, socialization, maintenance, role transition, and exit	Places volunteer careers within D4 while also linking them to D1, D3, D6, and D7

For each model, the table states what it primarily captures and what the present framework adds.

For each model, the table summarizes its primary focus and indicates the additional conceptual elements introduced by the present framework.

### 2.4 Continuum and asymmetry

The framework treats informal helping, structured volunteering, and high-cost helping as regions within a single continuous space rather than as discrete categories. This is a deliberate scope choice. It does not imply that these forms of engagement are psychologically identical. Rather, the continuity claim is that the same seven dimensions can organize the analysis of volunteer engagement across a wide range of forms. Within this continuous space, different forms of helping may occupy very different positions. Everyday informal helping may involve relatively low personal cost, limited role identity, and weaker integration into the self. By contrast, high-cost helping may involve substantial sacrifice, stronger moral obligation, deeper identity integration, and a more explicit sense of meaning. The framework therefore allows low-cost and high-cost helping to be compared without assuming that they are the same kind of experience. The asymmetry claim refers to this shift in dimensional salience. As personal cost increases, D5, Moral Commitment, D4, Identity Integration, and D6, Meaning, become increasingly central to understanding the engagement. D1, Motivational Basis, becomes less central in relative salience. This does not mean that motivation is absent in high-cost helping. Rather, it means that the sustaining force of the engagement may depend less on initial motives and more on moral commitment, identity, and meaning. A marked asymmetry at the high-cost pole therefore reflects a change in dimensional salience within the framework, not necessarily the emergence of a wholly separate category of helping.

The boundary of volunteerism is also narrower than the boundary of prosocial behavior. Prosocial engagement is the broader domain. Volunteerism refers to a specific subset of prosocial engagement: action that is voluntary, directed beyond immediate private or familial obligation, not directly materially compensated, and socially meaningful or role-like in form. For this reason, activism, caregiving, civic participation, and informal helping may overlap with volunteerism, but they are not automatically identical to it. They fall within the framework only when they meet the relevant criteria. The framework should therefore be understood as a model of volunteerism within the broader prosocial domain, rather than as a replacement for theories of helping behavior in general.

## 3 Volunteerism: costs, benefits, and context

A growing body of research indicates that volunteering is associated with beneficial mental, social, and physical health outcomes, including higher wellbeing, lower distress, greater social connectedness, better functional health, and, in some longitudinal studies, lower mortality risk (21–24). More recent studies report associations with lower levels of depression and loneliness, particularly among older adults, alongside stronger perceived purpose and social integration (25, 26). Because the evidence base is largely observational, it is vulnerable to healthy-volunteer selection and reverse causation. Filges et al. (22) and Jenkinson et al. (23) therefore caution against strong causal inference; accordingly, benefit claims in this paper are framed as association unless supported by experimental evidence.

These outcomes are also not uniform; they appear to depend on motivation (D1), organizational support, role characteristics, and intensity of involvement (2, 27). Volunteering is best understood as a psychosocial process that may involve both meaningful benefits and significant personal costs (D2). Volunteers may invest substantial emotional energy, time, physical effort, and personal resources, while also experiencing role strain, stress, and exposure to emotionally challenging situations. More emotionally intensive forms of volunteering, particularly those involving trauma exposure, caregiving, crisis response, or sustained contact with suffering, have been associated with burnout, compassion fatigue, emotional exhaustion, and increased depressive symptoms (28). These are related but distinct constructs: burnout is a syndrome of emotional exhaustion, cynicism, and reduced efficacy; emotional exhaustion is the depletion component of burnout; and compassion fatigue refers to secondary traumatic stress arising from empathic engagement with others' suffering. The cost-to-consequence link (D2 to D7) is consistent with stress-process and job demands-resources models, in which chronic demands without sufficient coping resources may produce psychological depletion (28).

The link between D2 and D7 can also be framed through conservation of resources theory: high personal cost is consequential when it threatens or depletes valued emotional, social, material, or physical resources, whereas support and meaning may protect or rebuild those resources (29). Moral injury provides a complementary lens for high-D5 contexts, because volunteers may experience distress when they witness, participate in, or are unable to prevent events that violate deeply held moral commitments (30). Compassion fatigue, as originally developed in trauma-care contexts, is therefore treated here as one possible negative indicator of D7 rather than as identical to D2 (31).

The relevant question is therefore not whether volunteering is inherently beneficial or harmful, but under what conditions it is associated with positive or negative outcomes. Several reviews suggest the volunteering-health relationship may be nonlinear, with moderate engagement often associated with more favorable outcomes than either minimal or excessively intensive involvement (32). Evidence for a precise optimal level remains inconsistent, and this inconsistency is itself a finding the framework must accommodate rather than resolve by assertion (23). The effects of volunteering appear to depend not only on the extent of involvement but also on the quality of the experience, the availability of support and coping resources, and the broader social and organizational context (D3).

Volunteers interpret their roles, appraise demands, evaluate recognition, and situate their actions within frameworks of meaning (D6) and identity (D4). Their experiences are also shaped by organizational conditions, including role clarity, training, supervision, perceived appreciation, and social support. The cost of volunteering (D2) cannot be reduced to time investment alone; it emerges through the interaction of emotional demands, intensity, subjective meaning, and available coping resources, and it issues in a psychological consequence (D7) that organizational support and perceived recognition appear to buffer (21, 23, 33). Volunteers are not passive providers of assistance but active meaning-making agents whose experiences are influenced by autonomy, perceived efficacy, social connectedness, and the alignment between personal values and organizational goals. Sustained, psychologically beneficial engagement is more likely when volunteering satisfies emotional, relational, and existential needs while remaining supported by adequate organizational and interpersonal resources. One mechanism for the favorable pole of psychological consequence (D7) is the positive-affect pathway described by broaden-and-build theory, on which the positive emotions generated by meaningful helping broaden cognition and build durable personal and social resources over time (34); this gives the meaning (D6) and consequence (D7) dimensions a defined affective mechanism rather than treating their benefits as assumed.

## 4 High-cost helping as dimensional convergence

The framework becomes most informative when applied to high-cost helping, in which volunteers incur substantial personal risks or sacrifices to benefit others. Examples include distributing food during wartime despite ongoing security threats, hosting displaced strangers after armed attacks or natural disasters, entering dangerous environments to rescue victims, or providing humanitarian aid in conflict zones and epidemic outbreaks. In the most extreme cases, individuals knowingly risk their lives to save others. Such acts are often treated as paradigmatic examples of altruism, yet they are difficult to accommodate within a one-dimensional altruism-maximization account, in which extreme helping is simply the upper end of a single altruism continuum and helping intensity tracks the strength of other-concern alone.

The framework offers a configural alternative. High-cost helping is predicted to exhibit a distinctive profile characterized by elevated personal cost (D2), moral commitment (D5), identity integration (D4), and meaning (D6), together with low anticipated reward within the motivational domain (D1). It is the overall configuration, rather than the level of any single dimension, that distinguishes high-cost helping from intense but otherwise ordinary volunteering. Alternative accounts, center on altruistic motivation alone imply a different classification logic. For empirical purposes, high-cost helping should be defined prospectively rather than retrospectively. A case would be classified as high-cost when it exceeds a preregistered threshold on a composite of time burden, emotional labor, opportunity cost, exposure to danger, financial cost, and responsibility for vulnerable recipients. The threshold should be context-specific but explicit, for example the upper quartile of a role-specific cost index or a score above a predetermined standardized cutoff. This prevents the label high-cost from being assigned only after a heroic or socially admired outcome is known. On the altruism-maximization account, the defining feature of extreme helping is the strength of other-concern; cost and anticipated reward are secondary. By contrast, the present framework predicts a specific multidimensional pattern. The critical question is therefore not whether highly costly helping occurs, but whether those who engage in it display the predicted configuration of dimensions.

The framework is empirically vulnerable in several ways. It would be disconfirmed if genuine high-cost helpers routinely reported substantial anticipated reward, if they scored no higher on moral commitment and identity integration than matched ordinary volunteers once cost was equated, or if predicted distinctions between dimensions failed to appear. The framework also predicts that dimensions need not always move together. For example, individuals who undertake costly actions on behalf of a highly valued in-group may show high identity integration (D4) without the elevated meaning (D6) expected among helpers motivated by broader humanitarian commitments. Failure to observe such divergences would count against the configural account. The framework is therefore prospectively falsifiable because its central claims generate empirical predictions that can be tested directly. Research in social psychology suggests that extreme helping is shaped by moral identity, internalized norms, perceived responsibility, situational immediacy, and identification with others (35, 36). Under such conditions, action may not be experienced as a calculated sacrifice but as the only acceptable response within a moral or relational frame (37) interviews with rescuers provide particularly strong evidence for this non-deliberative quality: rescuers commonly reported no experience of choice, describing a sense of shared humanity that rendered action self-evident rather than the product of cost-benefit deliberation. A volunteer who risks death entering a collapsed building after an earthquake is difficult to explain in terms of reciprocity or strategic advantage. The configural profile, high D2, D4, D5, and D6 combined with low anticipated reward, locates such acts within a broader pattern and predicts the absence of reward tracking that signaling accounts would expect. Heroism science is relevant here because it distinguishes heroic action from ordinary altruism by emphasizing voluntary action under conditions of risk or sacrifice on behalf of others or a moral cause (38). In the present framework, heroism is not added as an eighth dimension; rather, heroic acts are treated as one possible high-cost configuration, usually involving high D2, D4, D5, and D6 and variable D3 and D7. Several existing theories illuminate aspects of these cases. Identity fusion theory explains extreme self-sacrifice through a visceral sense of oneness with a group (39, 40), costly-signaling accounts interpret helping as the advertisement of desirable qualities (41), and evolutionary theories emphasize kinship and reciprocity (42, 43). Dual-process models suggest that intuitive processing may facilitate cooperation under conditions of immediacy (44). The present framework is not intended to replace these explanations. Rather, it provides a structure within which they can be located. Fusion may be one route to elevated identity integration (D4), signaling accounts make predictions about anticipated reward, and dual-process theories help explain how different configurations of prosocial engagement may arise through intuitive and deliberative pathways. The distinctive contribution of the proposed framework is that it predicts a configuration of dimensions. Competing theories may account for components of that configuration; the framework specifies how those components combine within episodes of prosocial engagement.

High-cost acts therefore occupy a region of the multidimensional space rather than constituting a separate category of behavior. Whereas ordinary volunteering typically involves a balance between contribution and manageable burden, high-cost helping is characterized by a marked asymmetry in which personal cost (D2) substantially exceeds direct benefit to the helper. The same seven dimensions remain operative, but their relative salience shifts: moral commitment (D5), identity integration (D4), and meaning (D6) become increasingly central, whereas anticipated personal benefit plays a diminished explanatory role. This illustrates the continuity-with-asymmetry position described in Section 2.4 in the form of a concrete profile. The public-health implications are direct. The dimensions that sustain engagement under extreme conditions are also those that, when unsupported, may increase vulnerability to moral injury, emotional exhaustion, compassion fatigue, and burnout. Consequently, the dimensions most characteristic of high-cost helping are also the dimensions most in need of organizational recognition, institutional support, and societal protection.

## 5 Volunteering and the limits of the altruism account

The discussion of high-cost helping raises an important theoretical question: how should such acts be understood in relation to altruism? Individuals who undertake demanding forms of helping often report emotional gratification, social appreciation, identity affirmation, meaning, and a sense of belonging. The presence of these benefits has long complicated attempts to define altruism, giving rise to debates about whether helping can ever be genuinely other-regarding when it may also provide rewards to the helper. This issue is reflected in the distinction among outcome-based, motive-based, and norm-based conceptions of altruism, which emphasize respectively the consequences of an action, the individual's intentions, and the social or moral norms that guide behavior (12).

The present framework does not seek to resolve these debates. Rather, it uses them as analytic perspectives for understanding high-cost helping. Consistent with the behavioral definition developed in Section 1, altruism is treated as one possible motivational basis for prosocial engagement (D1) rather than as a separate category of action. The relevant question is therefore not whether an act is truly altruistic, but how different conceptions of altruism relate to the broader pattern of dimensions that characterize the act. Consider an aid worker who knowingly enters a region affected by a highly contagious epidemic. From an outcome-based perspective, the act is altruistic because it benefits others despite substantial personal risk. From a motive-based perspective, the critical issue is whether concern for others rather than anticipated reward drives the behavior. From a norm-based perspective, the act reflects an internalized sense of moral obligation. Each interpretation captures part of the phenomenon. The framework, however, predicts a broader pattern: high personal cost (D2), strong moral commitment (D5), elevated identity integration (D4), strong meaning (D6), and low anticipated reward. The explanatory claim therefore concerns the combination of dimensions rather than any single criterion of altruism.

Although this example involves an extreme form of helping, the same logic applies across the broader continuum of volunteering. Similar helping behaviors may emerge from different combinations of motives, meanings, identities, and obligations. What distinguishes forms of engagement within the framework is not simply whether they are altruistic, but the pattern of dimensions through which they are expressed. The same reasoning applies to acts such as rescuing victims from a collapsed building after an earthquake. The significance of such actions cannot be captured fully by reference to altruistic motivation alone. Rather, they reflect the convergence of multiple dimensions, including personal sacrifice, moral commitment, identity, and meaning. This broader perspective also helps explain why individuals facing similar risks may differ substantially in their willingness to act.

The framework therefore remains neutral regarding the existence of psychologically pure altruism. It neither requires pure altruism nor treats it as the hidden explanation of high-cost helping. Instead, it characterizes prosocial engagement through a multidimensional structure within which altruistic motives may play an important, but not exclusive, role.

Rather than classifying high-cost helping according to the presumed purity of altruistic motivation, the framework shifts attention toward the psychological and social processes that sustain prosocial engagement under demanding conditions. In doing so, it offers a broader account of volunteering that accommodates both ordinary and extraordinary forms of helping within the same conceptual space.

## 6 Moral commitment, meaning, and the cross-disciplinary borrowings

Moral commitment (D5), identity integration (D4), and meaning (D6) draw on distinct traditions and must perform different explanatory functions. D5 concerns commitment to moral values and obligations, D4 concerns the incorporation of helping into the self-concept, and D6 concerns purpose and meaning. Moral identity theory (45) links D5 and D4 because acting prosaically may express core values rather than instrumental calculation (46). However, moral commitment is not reducible to identity. People may experience a strong moral obligation to help without defining themselves through helping, just as helping may be self-defining without being experienced as meaningful.

The expected empirical relationship among D4, D5, and D6 is therefore correlation rather than interchangeability. D5 may precede action as a felt obligation, even before a volunteer role becomes self-defining. D4 may increase gradually as repeated participation becomes incorporated into biography and self-concept. D6 may arise when the activity is appraised as purposeful or coherent within a larger life narrative. These temporal and phenomenological differences generate testable discriminant predictions: identity items should load primarily on D4, obligation/value-binding items on D5, and purpose/coherence items on D6. If these indicators collapse into one undifferentiated factor, the framework's distinctness claim would require revision.

Schwartz's theory of basic human values (47, 48) specifies which value types load on moral commitment. Benevolence, understood as concern for the welfare of close others or the in-group, and universalism, understood as concern extended to all people, are the value types the framework predicts at high D5. This distinction is important because the benevolence–universalism contrast is exactly what differentiates high-cost helping directed primarily toward one's own group from high-cost helping motivated by concern for all people, as discussed in Section 8.

Frankl's logotherapy and Park's meaning-making model (19, 20) anchor D6 specifically in the will to meaning and in the human capacity to find purpose despite, and even through, suffering. This formulation makes D6 conceptually distinct from the self-definition that anchors D4. An act can be self-defining without being experienced as meaningful in Frankl's sense, and it can be meaningful without being self-defining (37) phenomenology of rescuers supplies the central evidence that D5 can operate non-deliberatively. Her interviewees reported that they perceived no real choice, a finding that is used here not as a parenthetical citation but as direct support for the framework's claim that, under conditions of immediacy, moral commitment may be experienced as imperative rather than as deliberation.

Social identity and self-categorization theories (49), Ellemers et al. (50) supply the self-group mechanism for D4 in cases of self-sacrifice. This mechanism should be distinguished from role identity theory (8), which concerns identity attached to a social role. The framework therefore uses role identity for ordinary role-based volunteering, and social identity, including its fusion variant, for high-cost self-sacrifice. In this way, the framework draws on both traditions without conflating them.

A distinction therefore emerges between ordinary volunteering and helping under acute threat. Both may be motivated by concern for others and by values, but they differ in the configuration of cost, social embeddedness, and expected benefit. Ordinary volunteering is embedded in ongoing social contexts (D3) that also provide the individual with psychological and social benefits, including wellbeing, connectedness, purpose, and belonging. Helping therefore occurs within systems of mutual reinforcement and moderate cost. Helping during war, disaster, or epidemic involves substantial cost (D2), often with little realistic prospect of reciprocal benefit. It is therefore more reflective of moral commitment (D5) and meaning (D6) that remain salient even when circumstances constrain deliberation (19, 36, 37). In these cases, the focus shifts from anticipated outcomes to alignment with values, identity, and meaning. This places acute-threat helping in a high-D5, high-D6 region of the framework's conceptual space.

## 7 Propositions and a measurement program

The framework yields five falsifiable propositions; each paired with an empirical test.

Proposition 1: Dimensional structure. Indicators of D1–D7 will resolve into seven distinct but correlated latent factors, with no dimension empirically redundant with another.

Proposition 2: Nonlinear dose–response. The association between engagement intensity and favorable psychological consequence (D7) is nonlinear, with moderate involvement associated with better outcomes than either minimal or excessive involvement.

Proposition 3: Configural account of high-cost helping. High-cost helping is characterized by the joint elevation of personal cost (D2), moral commitment (D5), identity integration (D4), and meaning (D6), together with low anticipated reward. The framework predicts that this configuration will distinguish high-cost helpers from otherwise comparable volunteers more effectively than any single dimension alone.

Proposition 4: Organizational-support buffering. Organizational support moderates the association between personal cost (D2) and psychological consequence (D7), such that the cost-to-burnout association is weaker under conditions of high support.

Proposition 5: Motivation-by-meaning interaction. The contribution of meaning (D6) to favorable consequence (D7) is greater when motivational basis (D1) is more other-oriented, such that meaning buffers personal cost most strongly among value-driven volunteers.

The measurement program is feasible because the framework specifies a fixed dimensional structure. Accordingly, the first empirical task is to test whether the seven theorized dimensions can be recovered as distinguishable but correlated latent factors. Each dimension should be represented by multiple indicators: D1, validated volunteer-motivation measures such as the Volunteer Functions Inventory (5), with separate items for anticipated reward; D2, time commitment, financial outlay, emotional labor, opportunity cost, responsibility for vulnerable recipients, and exposure to danger; D3, role formality, organizational mediation, and network embeddedness; D4, identity centrality and identity fusion; D5, moral identity and internalized obligation; D6, meaning and purpose; and D7, wellbeing, connectedness, burnout, compassion fatigue, and distress. Where possible, indicators should be drawn from established and validated measures in the volunteering, identity, morality, meaning, and wellbeing literatures.

Anticipated reward within D1 should be operationalized as a prospective expectancy rather than an outcome. It should distinguish anticipated social recognition, anticipated emotional satisfaction, instrumental or self-development benefit, anticipated reciprocity, and other-oriented concern. These facets separate anticipated reward from D3, which concerns social or organizational structure, and from D7, which concerns actual psychological consequences after engagement. This distinction is essential because otherwise anticipated reward could be confused with either the social context of volunteering or its later psychological effects.

Because D7 is an outcome dimension, the strongest empirical tests should use longitudinal or repeated-measures designs. At baseline, D1–D6 can be assessed as predictors of later D7; over time, D7 can also feed back into motivation, identity, and meaning. This temporal modeling addresses the concern that wellbeing, burnout, and growth are usually consequences rather than antecedent features of volunteering, while preserving their role in the framework as part of the complete volunteer-engagement profile.

The level of analysis should be specified in advance. Episodes of helping provide the clearest unit for D2 and immediate D7; roles provide the clearest unit for D3 and role-related D4; persons provide the clearest unit for stable motivational, identity, moral, and meaning tendencies; and organizations provide the clearest unit for support, supervision, role clarity, and duty-of-care conditions. Empirical applications should therefore use multilevel designs where possible, with episodes nested within persons and persons nested within organizations. Common-method bias should be reduced by time-separated measurement, objective indicators such as time logs or exposure records, organizational reports of role characteristics, and independent measures of wellbeing or burnout (51).

Sample design should follow the purpose of each empirical test. For initial scale development, the study should include enough participants to estimate the factor structure reliably. A practical starting point is at least ten participants for each proposed item. The first step should be exploratory factor analysis (EFA), to examine whether the proposed indicators organize into the expected dimensions. The second step should be confirmatory factor analysis (CFA), to test whether the hypothesized seven-factor structure provides an adequate fit to the data. When feasible, EFA and CFA should be conducted in separate samples, with approximately 300 to 500 participants in each sample. Because high-cost helping is relatively rare, validation studies should intentionally include larger numbers of volunteers from crisis intervention, caregiving, emergency services, humanitarian aid, and disaster response, while also retaining comparison groups of ordinary volunteers. Any changes to the measurement model should be planned and preregistered. The study should specify clear rules for when model revision will stop, and any revised model should be tested again in an independent sample using CFA. Finally, measurement invariance should be examined across age, gender, socioeconomic position, role type, and cultural context. Before comparing latent means across groups, multi-group confirmatory factor analysis should test whether the same factor structure, factor loadings, and item intercepts are sufficiently similar across groups, corresponding to configural, metric, and scalar invariance (52).

For the specific risk of overlap among identity integration (D4), moral commitment (D5), and meaning (D6), CFA results should be treated as a decision point. If these dimensions show high intercorrelations, weak discriminant validity, or partial overlap, the seven-factor CFA model should be compared with theoretically plausible alternatives, including bifactor and network models. A bifactor model would test whether a general prosocial-engagement factor coexists with specific residual factors for D4, D5, and D6, whereas a network approach would examine whether identity, obligation and value binding, and purpose and coherence indicators form separable empirical clusters rather than one undifferentiated construct (53). The configural propositions can be preserved only if meaningful dimension-specific variance remains. If D4, D5, and D6 collapse completely across samples, contexts, and methods, the framework should be revised rather than defended. This decision rule makes the distinctness claim falsifiable rather than protected from contrary evidence.

For Proposition 2, nonlinear dose-response relationships should be tested prospectively by including quadratic terms and, where sample size allows, spline-based or generalized additive models. The central empirical question is whether engagement follows an inverted-U pattern, such that moderate levels of involvement are associated with the most favorable D7 profile. Models should adjust for baseline health, prior volunteering experience, role type, socioeconomic resources, and organizational support. To reduce the risk of reverse causality, future studies should use longitudinal cross-lagged or fixed-effects designs, repeated assessments before and after changes in role intensity, and, where available, quasi-experimental sources of variation, such as organizational redeployment, crisis onset, or changes in service demand.

For Proposition 3, future studies should define high-cost helping in advance using a preregistered cost index. This index should include several types of cost: time burden, emotional labor, opportunity cost, exposure to danger, financial cost, and responsibility for vulnerable recipients. Researchers should then test whether profile-based models explain high-cost helping better than simpler models based only on separate costs or statistical interactions. Model comparison should examine overall model fit, classification quality, explained variance, prediction error, and whether the findings remain stable when different cut-offs for high-cost helping are used. To reduce selection and survivorship bias, studies should measure participants' baseline health and prior volunteering, including people who left high-cost roles, and, where possible, use propensity-score or matched comparison designs. Retrospective narratives may also be useful, but they should be interpreted cautiously because they may overrepresent people who remained in these roles or found them meaningful.

For Propositions 4 and 5, future studies should specify the SEM moderation strategy in detail. This includes reporting how variables are centered, scaled, and coded; how interaction terms are constructed; and how latent interactions are estimated when used. Organizational support should be measured with validated indicators, including supervision, training, role clarity, peer support, and perceived organizational support. D2 should be triangulated with objective indicators such as hours, exposure, and caseload intensity. D7 should be assessed with validated measures of wellbeing, burnout, compassion fatigue, and distress, with physiological or administrative indicators added where feasible. The key test for Proposition 4 is the D2 × organizational support interaction predicting burnout or distress. The key test for Proposition 5 is the D1 × D6 interaction predicting favorable D7 outcomes.

Qualitative narrative interviews with volunteers, caregivers, and first responders could complement the quantitative research program. These interviews would examine how people involved in high-cost helping understand the costs of their roles and how they integrate such roles into identity, morality, and meaning. They would not replace quantitative tests of the propositions but would help clarify how helpers themselves distinguish between cost, obligation, identity, and meaning in lived experience.

## 8 Implications for public health and practice: from dimensions to levers

The framework suggests that volunteering is not uniformly health-promoting but a context-dependent form of engagement whose psychological consequences (D7) depend on the configuration of the other dimensions. Its practical value lies in translating these dimensions into specific organizational levers rather than offering a generic call for support.

Motivational basis (D1) implies attention to recruitment, selection, and retention strategies. Because volunteers enter roles for different reasons, including altruistic concern, social connection, personal growth, identity expression, and moral commitment, organizations should use recruitment and retention approaches that resonate with these differing motivations. Tailoring opportunities to volunteers' motivational profiles may enhance engagement and persistence. Personal cost (D2) points to graded protection, including supervision, rotation, exposure limits, psychological first aid, and debriefing calibrated to the demands of the role. Social embeddedness (D3) highlights the importance of role clarity, peer support, team integration, and organizational structures that foster social connection and reduce isolation. Identity integration (D4) suggests the value of enabling volunteers to incorporate their role into a coherent sense of self. Opportunities for autonomy, skill development, and long-term role continuity may strengthen this integration and support sustained commitment. Moral commitment under acute threat (D5) requires duty-of-care protections, insurance, clear ethical guidance, and attention to moral distress in roles that involve difficult or high-stakes decisions. Meaning (D6) implies that programs should help volunteers understand how their activities contribute to broader goals and collective outcomes, thereby reinforcing a sense of purpose beyond the immediate task. Psychological consequence (D7) calls for routine monitoring of wellbeing, burnout, distress, and growth outcomes alongside participation indicators.

Screening and matching cut across these dimensions: assigning volunteers to roles that fit their resources, motivations, and vulnerabilities is a primary preventive strategy, and the buffering predicted in Proposition 4 provides one mechanism through which such matching may operate. These mappings are summarized in Table 3.

**Table 3:** Mapping each canonical dimension to a public-health lever (Section 8).

Dimension	Public-health lever
D1 Motivational basis	Recruitment messaging, volunteer selection, and motive-aware role matching
D2 Personal cost	Supervision intensity, rotation, exposure limits, psychological first aid, and debriefing
D3 Social embeddedness	Role clarity, structured peer support, team integration, and organizational mediation
D4 Identity integration	Recognition practices and role crafting that support alignment between volunteering and self-defining commitments
D5 Moral commitment	Duty-of-care protections, insurance, ethical guidance, and moral-distress safeguards
D6 Meaning	Connecting tasks to a credible larger purpose and communicating program impact
D7 Psychological consequence	Routine monitoring of wellbeing, burnout, distress, and positive psychological outcomes

Proposed mappings are theoretically derived and have not yet been empirically evaluated.

These distinctions are relevant for public-health systems that rely on volunteers in crisis response, caregiving, health promotion, and psychosocial support. Volunteers should be understood not only as contributors to service delivery but also as participants whose wellbeing requires ongoing organizational attention. Moving beyond narrow indicators such as participation frequency or hours volunteered toward multidimensional assessments that incorporate motivation, cost, social embeddedness, identity, meaning, organizational context, and psychological consequences may provide programs with better information for prevention, support, and role design.

At the societal level, volunteering may strengthen social cohesion, collective resilience, and shared meaning during adversity, consistent with the social-capital tradition linking civic participation to community resources (54). At the same time, reliance on volunteers raises questions about the distribution of costs and responsibilities. Emotionally demanding roles are not distributed evenly and may be more common among women, older adults, and economically precarious groups. Moreover, when volunteer effort compensates for limitations in formal institutional responses, some responsibilities that would otherwise be carried by professional services may shift toward citizens and community networks. The framework therefore suggests that assessments of volunteer engagement should consider not only its benefits but also who bears its demands and how volunteers are supported. Volunteering is best understood as a complement to, rather than a substitute for, adequately resourced professional services, particularly during prolonged emergencies, disasters, and trauma-related crises.

Safeguards are needed because the same high-D5 and high-D6 profile that supports sustained engagement may also increase vulnerability to excessive demands. Moral commitment, identity, and meaning should not be assumed to justify unsafe work, prolonged exposure, insufficient training, or the substitution of unpaid labor for professional services. Duty of care should therefore include clear role boundaries, informed consent about risks, the possibility of opting out without stigma, supervision, insurance or liability protection, rotation and rest, escalation routes to professional services, monitoring of distress, and explicit limits on what volunteers may be asked to do. In high-risk roles, strong commitment should be accompanied by stronger organizational protection.

The life-course and social-inequality implications of volunteering also require careful consideration. Access to volunteering, and exposure to high-cost roles, are shaped by socioeconomic resources, time poverty, caregiving obligations, gendered and ethnicized expectations of care, age, disability, migration status, and proximity to crisis. Low participation should therefore not be interpreted only as low motivation or weak civic commitment. Conversely, high participation in demanding roles may reflect structural necessity, social expectation, or unequal burden as well as freely chosen civic engagement. Public-health applications should therefore examine both the potential benefits of volunteering and the emotional, economic, and opportunity costs associated with it. A multidimensional framework can help identify these differences and support more equitable role design, supervision, and protection.

## 9 Limitations and future research

Several limitations should be acknowledged. First, the framework is theoretical and requires empirical validation. The seven dimensions have not yet been operationalized within a single measurement model, although Section 7 specifies how such a validation program could proceed and what kinds of findings would challenge the proposed structure. Prosocial behaviors are shaped by cultural, social, organizational, and historical circumstances. The dimensions should therefore be understood as a defined but revisable basis for measurement, rather than as fixed or universal categories. Future empirical work may reveal stronger overlap among some dimensions than anticipated, particularly identity integration (D4), moral commitment (D5), and meaning (D6). Such findings would require refinement of the proposed structure and, where necessary, revision of the boundaries among these dimensions.

A further limitation is that the manuscript offers hypotheses and propositions rather than empirical results. Consequently, claims about the validity of the seven-dimensional structure, the distinctness of D4–D6, and the configural account of high-cost helping should be read as theoretically grounded and prospectively testable, not as empirically demonstrated in the present article. The paper's interpretation of existing research is integrative and hypothesis-generating. Future studies should examine whether the proposed configuration improves prediction beyond existing motivational, identity-based, meaning-based, and organizational models of volunteering and helping.

The boundaries among informal helping, structured volunteering, and high-cost helping are also difficult to define with precision. They are best understood as regions of a continuum rather than as discrete categories. Individuals may move across these regions over time, and the same activity may acquire different meanings depending on context, duration, perceived obligation, organizational embedding, and personal cost. In addition, volunteers' motives are often complex, evolving, and only partly accessible to reflection. A person may describe an action in terms of altruism, obligation, identity, reciprocity, faith, political commitment, or personal meaning, and these accounts may coexist rather than compete. The framework therefore does not assume that motives can always be classified cleanly or reduced to a single dominant source.

High-cost helping presents additional methodological challenges. It is relatively rare, often context-specific, and frequently studied through retrospective accounts, historical documentation, testimony, organizational records, or collective memory. These sources are valuable, but they may be affected by selection, survivorship, social desirability, narrative reconstruction, and the moral framing of events after they have occurred. Consequently, interpretations of motive, cost, identity, and meaning remain constrained by the available evidence. The vignette and factorial-survey designs proposed in Section 7 are intended partly to provide experimental leverage on phenomena that cannot easily be observed as they occur. Such designs cannot replace real-world observation, but they may help clarify how people evaluate cost, obligation, risk, recognition, organizational support, and moral commitment under controlled conditions.

The framework does not resolve the philosophical question of whether psychologically pure altruism exists, nor is it intended to do so. Its purpose is more limited: to provide a dimensional account of volunteering and high-cost helping that can be translated into empirical propositions. Future research should test the proposed dimensional structure across diverse cultural, organizational, and historical contexts, and examine when, for whom, and under what conditions volunteering promotes wellbeing, resilience, connectedness, and sustained engagement. Equally important, future research should examine when volunteering contributes to burden, distress, compassion fatigue, moral distress, or burnout. Public-health research would benefit from multidimensional assessments that incorporate emotional burden, meaning, identity, organizational context, role demands, and support, thereby providing a more comprehensive understanding of volunteer engagement and its consequences.

Future research may also use computational or ecological indicators, such as social-media discourse, digital traces, geographic indices, administrative records, or simulations. However, these indicators should be treated as contextual or hypothesis-generating data rather than as direct measures of volunteering prevalence or psychological consequence. For example, social-media activity may reflect visibility, expression propensity, platform access, media attention, or organizational communication more than actual helping behavior. Geographic or administrative indicators may capture opportunity structures, exposure to crisis, institutional capacity, or reporting practices rather than individual motivation. Simulation approaches could nevertheless be useful for examining how organizational policies, social embeddedness, resource constraints, and structural inequalities may shape different configurations of D1–D7. Used cautiously, such approaches may help generate hypotheses, identify system-level patterns, and guide the design of future empirical studies, while avoiding the assumption that digital or ecological traces are equivalent to volunteering itself.

## Data Availability

The original contributions presented in the study are included in the article/supplementary material, further inquiries can be directed to the corresponding author.
